# Core–Shell Chitosan Particles Targeting Membrane-Bound Heat Shock Protein 70 for Cancer Therapy

**DOI:** 10.3390/nano14231873

**Published:** 2024-11-22

**Authors:** Elena V. Svirshchevskaya, Valentina V. Kostenko, Anna A. Boyko, Maxim Shevtsov, Roman V. Kholodenko, Maria V. Grechikhina, Iuliia A. Gracheva, Alexey Yu. Fedorov, Alexander M. Sapozhnikov

**Affiliations:** 1 Laboratory of Cell Interactions, Department of Immunology, Shemyakin-Ovchinnikov Institute of Bioorganic Chemistry RAS, 117997 Moscow, Russia; office@ibch.ru (E.V.S.); amsap@mail.ru (A.M.S.); 2 Department of Radiation Oncology, Klinikum Rechts der Isar, Technical University of Munich, 81675 Munich, Germany; maxim.shevtsov@tum.de; 3 Laboratory of Biomedical Nanotechnologies, Institute of Cytology of the Russian Academy of Sciences (RAS), 194064 St. Petersburg, Russia; 4 Department of Organic Chemistry, Nizhni Novgorod State University, 603950 Nizhni Novgorod, Russia; yulia19gra4ova@gmail.com (I.A.G.); afedorovnn@yandex.ru (A.Y.F.)

**Keywords:** membrane-bound heat shock protein 70, targeting tumor cells, targeted therapy, chitosan core–shell nanoparticles, allocolchicinoid, 3D cultures

## Abstract

Anti-cancer targeted therapy is a promising approach. However, the identification of target molecules over-expressed in a wide range of tumors remains a significant challenge. The aim of this study was to analyze the expression of cell membrane-exposed heat shock protein 70 kDa (mHSP70) on different tumor cells and to develop a nanoscale delivery system based on a monoclonal antibody (mAb) that recognizes mHSP70 and uses chitosan core–shell nanoparticles (NPs). Several types of tumor cells (breast, pancreas, colon, prostate cancers, and some lymphomas) expressed mHSP70 as was determined by flow cytometry and confocal microscopy both in 2D and 3D cultures. Core NPs were formed by chitosan (C) conjugated to allocolchicinoid, which was used as a model drug (D). mAbs (A) targeting mHSP70 were complexed with succinylchitosan and used as NP shells forming final CAD-NPs. These NPs were characterized by size, charge, and functional activity. CAD-NPs were shown to have additional toxicity in comparison with CD-NPs in mHSP7-positive cells. Taken collectively, this study shows that mAb to mHSP70 can be used as a targeting vector in antitumor therapy.

## 1. Introduction

Cancer is a leading cause of death worldwide. Although there has been significant progress in recent decades in understanding the mechanisms of tumor transformation and development and their interaction with the immune system, most clinical approaches to treating cancer patients are still based on radio- and chemotherapy with or without adjuvant therapy. Targeted therapy using tumor-specific markers is currently showing great promise in improving the efficacy of cancer treatment [1,2]. Such markers may include but have not yet included members of the heat shock protein (HSP) family (e.g., HSP40, HSP60, HSP70, and HSP90) present on the surface of various types of solid and hematological malignancies [3,4,5]. There is now considerable evidence that many tumor cell types are characterized by the translocation of intracellular HSP70 to the surface of the plasma membrane (mHSP70), which can be used to target tumor cells. Although no regions in the structure of mHSP70 have been identified that could explain its binding to the plasma membrane, a number of studies have demonstrated its electrostatic and hydrophobic interaction with specific lipid peptides, including phosphatidylserine (PS) [6,7,8] and the glycosphingolipid globoyltriaosylceramide (Gb3) for lipid rafts [9,10]. 

One possible and promising approach to target the membrane-bound chaperone is to use monoclonal antibodies (mAbs) against mHSP70, which is present on the surface of tumor cells. It is well known that antibody preparations that selectively interact with cancer cells can be used for targeted antitumor immunotherapy [1,2]. HSP70 belongs to a large family of stress-induced proteins. One of the main functions of these molecules is to protect intracellular proteins from the damaging effects of various natural stress factors [11]. In addition, HSP70 plays an important role in the vital activity of cells under normal physiological conditions, interacting with a wide range of intracellular proteins and performing auxiliary, so-called chaperone functions. These functions of HSP70 are realized in the intracellular space, but in some cases, these proteins are also found on the cell surface and in the extracellular environment. In particular, mHSP70 is found on the surface of the tumor, virus, or parasite infected [12,13] and stressed cells [14]. We have previously shown that translocation of HSP70 to the cell surface is observed in cultures of tumor lymphoid cell lines [15]. The mechanisms of HSP70 translocation to the cell surface and secretion of these proteins into the extracellular space are poorly understood. It has been shown that HSP70 is transported to the cell surface by a non-classical mechanism independent of the Golgi apparatus. We have shown that the translocation of HSP70 to the cell surface increases during the final stages of apoptosis and is probably aimed at stabilizing the membranes of dying cells [15]. Many groups have also demonstrated the phenomenon of HSP70 secretion in cell cultures from various tissues [16]. The possibility of HSP70 exocytosis in vivo is suggested by data on the presence of HSP70 in blood serum, both normally and in various pathologies [17]. It is important to note that such an unusual extracellular localization of HSP70 activates the immune system. For example, it is now known that surface HSP70 stimulates the cytotoxic and migratory activities of natural killer (NK) CD94+ cells [3,18]. Extracellular HSP70 enhances cytokine production and accelerates the maturation of antigen-presenting dendritic cells [19]. Extracellular HSP70 found in tissues is considered by many authors to be an “alarm signal” for the immune system [20]. HSP70 has been shown to interact with a number of receptors on the surface of antigen-presenting cells, namely CD14, CD40, CD91, LOX-1, TLR-2, and TLR-4 [21]. The interaction of HSP70 with lymphoid cells is suggested by studies demonstrating the direct activating effect of these proteins on peripheral blood T lymphocytes in a number of autoimmune diseases [22]. Information on the direct activating effect of HSP70 on the complement system [23] is also of undoubted interest, suggesting the possibility of complement-dependent reception of these proteins by immune system cells. Our preliminary data suggest the possibility of receptor-independent internalization of extracellular HSP70 by lymphoid cells. Such a pathway of internalization of exogenous HSP70 may be related to the demonstrated ability of these proteins to interact with plasma membrane phospholipids [24]. Taken together, these data suggest an important but poorly understood role for extracellular HSP70 in the functioning of the immune system. The role of intracellular HSP70 translocation to the cell membrane surface and the mechanisms involved have not been elucidated. The conformational features of HSP70 associated with the surface of tumor cells have also not been characterized. It is precisely because of this circumstance that there are reasonable ideas that anti-mHSP70 targeting molecules, including monoclonal antibodies, antibody Fab fragments, and peptides directed against specific parts of the molecule of this protein, are necessary for the detection of mHSP70 [4,25]. The results of these experiments showed significant differences in the binding of our monoclonal antibodies to HSP70 expressed on the surface of a number of tumor cell lines.

Chitosan is a deacetylated derivative of the natural polymer chitin [26]. Chitosan is a biocompatible, nontoxic polycationic biopolymer easily modified due to the availability of multiple amino and hydroxyl groups in its structure [27,28]. Chitosan nanoparticles, gels, and scaffolds are often used as drug delivery systems [29]. We have previously used chitosan conjugated to allocolchicinoid and demonstrated its antitumor activity [30]. Natural alkaloid colchicine is a mitotic poison that binds to the intracellular protein tubulin and prevents the formation of the mitotic spindle. This leads to a block in cell proliferation and reduced cell motility. In clinical practice, it is used in low doses to treat gout, familial Mediterranean fever, amyloidosis, progressive systemic scleroderma, cirrhosis, and Behçet’s disease. Earlier, colchicine was attempted for the treatment of cancer and discontinued due to varying sensitivities in patients, but new derivatives of colchicine are currently being investigated [31,32,33]. For the preparation of chitosan–allocolchicinoid, a carboxyl group derivative was previously synthesized, which was also used as a model drug in this work. 

This paper aims to prove the concept of whether mHSP70 can be used as a target in antitumor therapy.

## 2. Materials and Methods

### 2.1. Materials 

Succinic anhydride (Pierce), 1-ethyl-3-[3-dimethylaminopropyl] N-(3-dimethylaminopropyl)-N′-ethylcarbodiimide hydrochloride (EDC) (Pierce), and N-hydroxysuccinimide (NHS) (Reanal Reanal Finechemicals, Budapest, Hungary) of analytical grade were used as received. Materials used: Sodium tripolyphosphate (Sigma-Aldrich, St. Louis, MO, USA), calcium chloride (Pacreac, Barcelona, Spain), acetic acid (Chimmed, Russia), ammonium hydroxide (Chimmed, Moscow, Russia), ethylenediaminetetraacetic acid (EDTA), (Sigma-Aldrich, USA), fetal bovine serum (FBS) (HyClone, Logan, UT, USA), RPMI 1640, phosphate-buffered saline (PBS), paraformaldehyde (Chimmed, Moscow, Russia), L-glutamine, ampicillin (all from PanEco, Moscow, Russia), fluorescein isothiocyanate (FITC) (Sigma-Aldrich, USA), Cyanine3-NHS (Lumiprobe, Moscow, Russia), Hoechst 33342 (Sigma-Aldrich, USA), 3-(4, 5-dimethyl-2-thiazolyl)-2, 5-diphenyl-2H-tetrazolium bromide (MTT, Sigma-Aldrich, USA).

#### Chitosan and Its Derivative

Chitosan (C) with a molecular weight (MW) of 30 kDa and a degree of deacetylation (DD) of 93% was prepared from C with an MW of 1040 kDa and DD of 85% (Aladdin Chemistry Co., Ltd., Shanghai, China) by chemical depolymerization using nitric acid as previously described [34]. N-succinyl chitosan with a degree of substitution of 75% was prepared from C with a MW of 30 kDa using succinic anhydride as previously described [35]. The MW was determined by high-performance liquid chromatography (GPC PSS NOVEMA Max analytical 1000 A column, PSS, Mainz, Germany). Pullulans («PSS», Germany) were used as calibration standards. 1H-NMR spectra of C and N-succinyl chitosan were recorded on a Bruker AMX 400 spectrometer (Bruker, Billerica, MA, USA). 4,4-Dimethyl-4-silapentane-sulfonic acid was used as a standard. Chitosans were purified by extensive dialysis.

### 2.2. Methods

#### 2.2.1. Antibody Production

Hybridomas to HSP70 fragments (2E4, 2E5, 2E11, 2F3, and 6G2) were donated by Vetchinin S.S. (State Research Center for Applied Microbiology and Biotechnology, Obolensk, Russia). Hybridoma cells were cultivated in RPMI-1640 supplemented with 10% fetal calf serum (FCS) pen-strep-glut (all from PanEco, Moscow, Russia). For antibody production, 2 × 10^6^ hybridoma cells were injected intraperitoneally into BALB/c mice pretreated with pristane (ICN Biochemicals, Cleveland, OH, USA) a week in advance (500 µL/mouse). Upon ascite formation, mice were sacrificed, and peritoneal fluid was collected. IgG fraction was purified by column chromatography on protein A sepharose (HiTrap, Sepharose FF rProtein A, Cytiva, Washington, DC, USA).

#### 2.2.2. Animals

All animal experiments were carried out according to the IBCh RAS IACUUC protocol. Female BALB/c mice (6–8 weeks) were obtained from the Stolbovaya animal farm (Moscow region, Russia). For 2 weeks, the mice were housed in plastic cages, with 10–12 per cage, under conventional minimal pathogen conditions. They were kept in a 12 h light/dark cycle at room temperature and fed ad libitum. The protocol was approved by the Institutional Animal Care and Use Committee (IACUC) of IBCh Animal Research Center (permit number 325 from 14 June 2021).

#### 2.2.3. Two-Dimensional and Three-Dimensional Cell Cultures

Human breast cancer cell lines BT474, BT20, MCF-7, and HCC1395; colorectal cells HT-29, HCC116; pancreatic cells PANC1; prostate cancer cells PC-3, Du145, lymphomas Raji, Daudi, K562, Jurkat and murine thymoma cell line EL-4 were used (all from Shemyakin-Ovchinnikov Institute of Bioorganic Chemistry RAS collection). Cells were grown in DMEM or RPMI-1640 media supplemented with 10% fetal calf serum (FCS) pen-strep-glut (all from PanEco, Moscow, Russia). Cells were incubated in CO_2_ at 37 °C. Cells were passaged by trypsinization using Trypsin/EDTA solution (PanEco, Moscow, Russia) twice a week. All cell lines were maintained at low passage numbers and routinely checked for mycoplasma by PCR. 

To prepare multicellular spheroids (3D cultures), 24-well plates (Corning, Glendale, CA, USA) were coated with poly (2-hydroxyethyl methacrylate (pHEMA) (Sigma, Merck KGaA, Darmstadt, Germany), dried, and used at request. Cells were grown onto pHEMA-coated plates in a complete culture medium. Overnight incubation resulted in the formation of multicellular spheroids (3D cultures). 

#### 2.2.4. Flow Cytometry 

The expression of mHSP70 was analyzed by flow cytometry. For this, epithelial cells were trypsinized and washed in a pre-warmed complete culture medium. Fluorochromes Cyanine3-NHS (Cy3) or FITC labeled antibody to HSP70 (clone REA349, Milnenyi Biotec, Germany) were added to the cells in complete medium, incubated for 1 h at 4 °C, washed in PBS and analyzed by flow cytometry using FACScan device (BD, USA). Isotypic control antibodies (Milnenyi Biotec, Bergisch Gladbach, Germany) were used to identify the specific binding of mAb. A total of 20,000 events were collected. The results were analyzed using FlowJo 10 software (BD, Franklin Lakes, NJ, USA). 

#### 2.2.5. Confocal Microscopy 

For confocal analysis, cells were grown overnight on sterile coverslips in 200 µL of complete culture medium in 6-well plates (Costar, Washington, WA, USA) (2D cultures) or 3D conditions using pHEMA coated plates. Antibodies to HSP70 (1:1000 dilution) or NPs (1:100 dilution) were added to the wells and cultivated for 24 h. Hoechst 33342 (Sigma) was used to visualize nuclei. After incubation, cells were fixed with 1% paraformaldehyde, washed, and polymerized with Mowiol 4.88 medium (Calbiochem, Nottingham, UK). Slides were analyzed using Eclipse TE2000 confocal microscope (Nikon, Tokyo, Japan). 

#### 2.2.6. Construction of the Delivery System Equipped with the Drug and Antibody to HSP70 

To develop a delivery system containing both the drug and the antibody to HSP70, we used core–shell format. Core particles were designed to contain the drug; the shell of the particles was equipped with the antibody. 

1. Core particles. Allocolchicinoids containing a carboxyl group were used as a model drug (further “D”). D was immobilized on chitosan (“C”), producing a CD conjugate. To this end, 30 µL from a 20 mM solution in DMSO (30.7 µg) of D was dissolved in 1 mL of distilled water. Upon shaking, EDC (1 mg) and NHS (0.5 mg) dissolved in 50 µL of water were added dropwise to D-COOH and kept for 1 h at 4 °C. Chitosan 30 kDa 0.2% (*w*/*v*) solutions were prepared in 0.4% acetic acid. To form CD conjugate, 4 mL of chitosan (2 mg/mL) was dropwise mixed with the activated D and kept at shaking 1 h for conjugation. NPs were formed by the ionotropic gelation method. Tripolyphosphate (TPP) solution (Sigma-Aldrich, Munich, Germany) (1.0%) was added dropwise under magnetic stirring at 30 rpm until opalescence occurred, which was estimated by a Specol 11 spectrophotometer (Carl Zeiss Jena, Germany) at 590 nm. The resulting C-NPs particle suspension was diluted 10 times in water and 2 times centrifuged at 700× *g* for 20 min in Ultracel-100K (Amicon Ultra, Millipore, Ireland) to remove bystander products. The concentrated NPs were re-suspended in 4 mL of PBS. 

2. Shell succinyl chitosan. In parallel, 2 mL (2 mg/mL, 4 mg in total) of N-succinyl chitosan, dissolved in PBS, was mixed with 2 mg of 2E4 antibody at shaking for 1 h. 

3. Final particles (“CAD-NPs”) were formed by mixing CD-NPs from step 1 and succinyl chitosan–antibody complex from step 2. Core–shell NPs were formed by electrostatic interaction between positively charged CD-NPs and negatively charged succinyl chitosan–antibody complex. As controls, several other NPs were generated (Table 1). To obtain Cyanine3-NHS or FITC labeled variants of NPs, 10 µL of the dyes were added to 200 µL of core NPs and incubated for 20 min. Fluorescent-labeled NPs were washed from free dyes, as described above. 

#### 2.2.7. Characterization of NPs and Dynamic Light Scattering (DLS)

The protein content of NPs was analyzed by BCA (ThermoScientific, Walthan, MA, USA). The quantity of D in NPs was measured by spectrophotometry (Beckman Coulter, Washington, DC, USA) using allocolchicinoid as standard.

The average diameter of NPs was determined using 90 Plus Particle Size Analyzer (Brookhaven, Holtsville, NY, USA) in water (25.0 +/− 0.1 °C) at a scattering angle of 90° and wavelength of 661 nm using Big Particle Sizing Software (90Plus Particles Sizing Software Vers. 4.20). The Zeta potential of NPs was determined in 10 mM KCl solution using identical Big Pal Zeta-Potential ver 5.78 analyzer hardware and software.

#### 2.2.8. Scanning Electron Microscopy (SEM)

Scanning electron microscopy (SEM) analysis was performed by depositing 1 µL of CAD-NPs diluted 1:50 in DI water on a clean silicon wafer. After drying at room temperature, the silicon wafer was placed on an SEM sample holder and analyzed by using Helios G4 PFIB (ThermoFisher Scientific, Waltham, MA, USA) at high voltage (5 kV) in secondary electron mode with 250,000× magnification.

#### 2.2.9. Cell Cycle Analysis 

The cell cycle was analyzed using propidium iodide-stained DNA. Cells were incubated with NPs for 72 h in 2D conditions, trypsinized, washed in ice-cold PBS, fixed by 70% cold ethanol, and left for 2 h at −20 °C. Thereafter, cells were washed twice in PBS, stained with 50 μg/mL of propidium iodide (Sigma-Aldrich, St. Louis, MO, USA) in PBS, 10 µg/mL of DNAse, and analyzed by flow cytometry using FACScan device (BD, Franklin Lakes, NJ, USA). The results were analyzed using FlowJo software. 

#### 2.2.10. MTT-Assay 

The cytotoxic effect of NPs was estimated by a standard 3-(4, 5-dimethyl-2-thiazolyl)-2, 5-diphenyl-2H-tetrazolium bromide (MTT) test. In short, different dilutions of NPs were prepared on a separate plate in 100 µL; 10^4^ cells per well were then added in 100 µL. Non-treated cells served as controls. Plates were incubated for 72 h. For the last 3 h, 250 µg/mL of MTT was added in 10 µL to each well. After the incubation, the culture medium was removed, and 100 µL dimethylsulfoxide was added to each well. Plates were incubated at shaking for 15 min to dissolve formazan. Optical density was read on spectrophotometer Titertek (UK) at 540 nm. Results were analyzed using the Excel package (Microsoft). The viability index was calculated as ODexperiment/ODcontrol, where OD was MTT optical density.

#### 2.2.11. Statistical Analysis

Statistical analysis was performed using Student’s *t*-test. Comparison values of *p* < 0.05 were considered statistically significant.

## 3. Results

### 3.1. Expression of HSP70 by Tumor Cells

**Figure 1:**
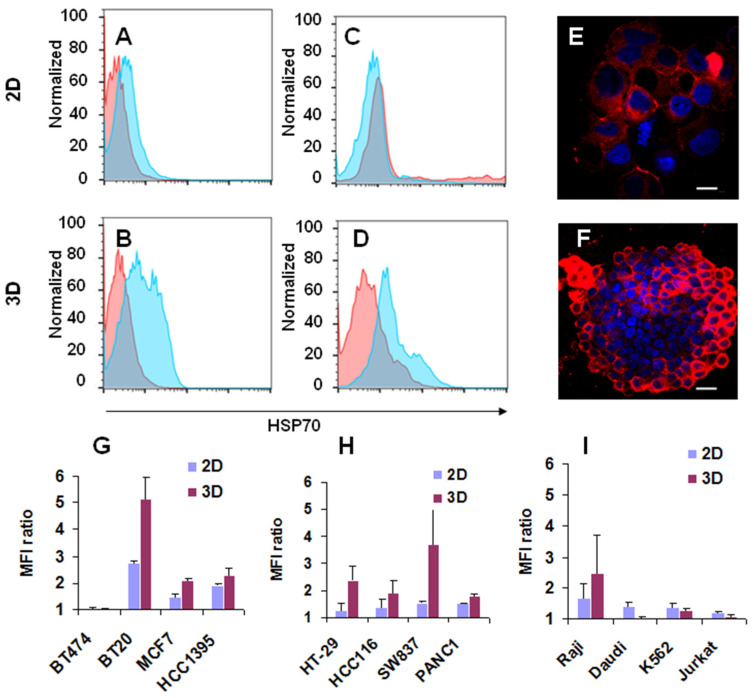
Expression of mHSP70 by different types of cells cultivated in 2D and 3D conditions. (**A**–**D**): Binding of anti-HSP70 antibodies to breast cancer BT20 (**A**,**B**) and colorectal cells HT-29 (**C**,**D**) incubated in 2D (**A**,**C**) or 3D (**B**,**D**) conditions analyzed by flow cytometry. Autofluorescence is shown in pink, and mHSP70 expression is shown in blue. (**E**,**F**): Confocal images of BT20 cells incubated in 2D (**E**) or 3D (**F**) conditions. Nuclei are stained in blue, scale bar 30 µm. (**G**–**I**): Ratios of mean fluorescence intensity (MFI) of anti-HSP70 labeled cell to isotype control MFI for different cells incubated in 2D and 3D conditions.

Cells of different origins were characterized by the expression of mHSP70 using commercial antibodies to HSP70. To model tumor growth in vivo, 3D cultures were compared with 2D ones. Several breast cancer cell lines (BT-474, BT20, MCF-7, HCC1395), colorectal (HT-29, HCC116, SW837), pancreatic (PANC1), prostate cancer cells (PC-3, Du145), lymphomas (Raji, Daudi, K562, EL-4) were analyzed. Many cells do express mHSP70 on the cell membranes (Figure 1A,G); others (BT474, HT-29, HCC116, Daudi, K562, EL-4) are almost negative in 2D cultures (Figure 1C,G–I). Many, but not all, started their expression in 3D conditions (Figure 1). Confocal microscopy also supported these results, as documented by brighter staining in 3D versus 2D cultures (Figure 1E,F). Of note, more HSP70 was translocated on the outer layer of the spheroids (Figure 1F). Lymphomas were mostly negative, However, Raji cells were positive both in 2D and 3D cultures (Figure 1I). These results indicate that HSP70 can be used as a targeting vector to treat different types of cancer.

### 3.2. Trans-Membrane Orientation of HSP70 

**Figure 2:**
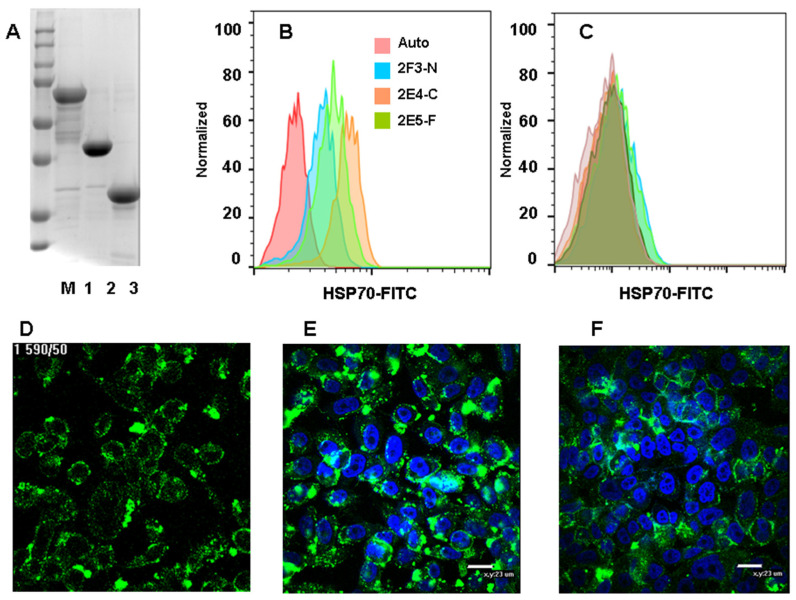
Orientation of mHSP70 on the plasma membrane. (**A**): Gel electrophoresis of HSP70 fragments used for antibody production. M- marker, full length (1), N-fragment (2), and C-fragment (3) of HSP-70. (**B**,**C**): Recognition of HSP70 by antibodies to full length (HSP70-F), C-(HSP70-C), or N-fragments (HSP70-C) on PC-3 (**B**) or Daudi (**C**). Autofluorescence is shown in pink, HSP70 expression—in different colors. (**D**–**F**): Confocal microscopy of PC-3 cells stained by FITC-labeled antibodies (green) to full length (**D**), C-fragment (**E**) or N-fragments (**F**). Nuclei are stained in blue, scale bar—23 µm.

The role of intracellular HSP70 translocation to the surface of the cell membrane and its mechanisms have not been deciphered to date. The conformational features of HSP70, associated with the surface of tumor cells, also were not characterized earlier. To this end, we used antibodies to full, N-, and C-fragments of HSP70. Among five hybridomas available, 2F3 recognized full-length HSP70 (Figure 2A, line 1), 2E5, and 6G2—N-fragment (line 2); and 2E4 and 2E11—C fragment (line 3). The titers of each IgG were more than 300,000. Analysis of antibody binding to mHSP70 positive cells PC-3 and negative ones Daudi demonstrated that antibody to the C fragment showed the highest fluorescence on PC-3 and did not bind the negative cells (Figure 2B,C). The antibody to N-fragment also bound PC-3 However, at a lesser level. These results evidenced that mHSP70 orientation on the membrane is stochastic, with substrate binding C-domain more exposed than the regulatory one N-domain. As soon as the cells before flow cytometry were treated with trypsin, which can cut off or truncate some proteins from the membranes, antibodies to HSP70 fragments binding to PC-3 cells were also analyzed by confocal microscopy. It appeared that anti-C fragment antibody 2E4 bound better than the others to full-length 2F3 or N-fragment (Figure 2D–F).

### 3.3. Development of Core–Shell Delivery System Targeting mHSP70 C Fragment 

**Figure 3:**
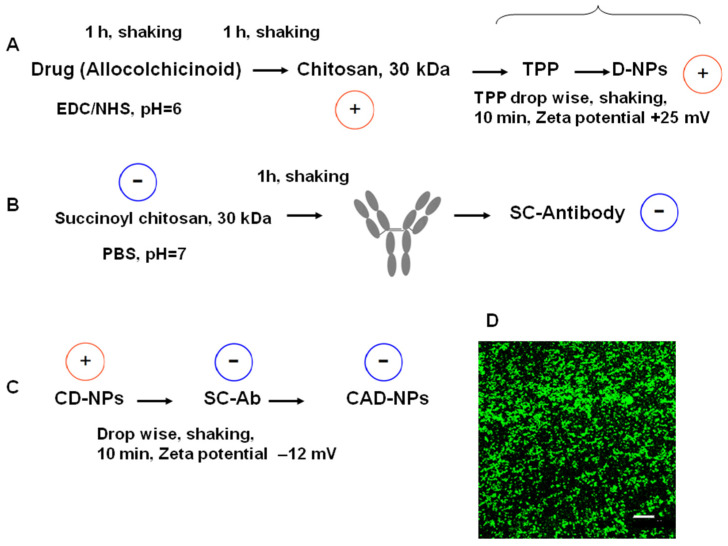
Scheme of the delivery system synthesis. (**A**): Particles were formed by sodium tripolyphosphates from allocolchicinoid (drug) activated by EDC/NHS and conjugated to chitosan 30 kDa (**C**). (**B**): Anti-HSP70-C fragment antibody (Ab) 2E4 was mixed with succinoylchitosan (SC), forming the complex via electrostatic interaction. (**C**): Final particles (CAD-NPs) were formed by electrostatic interaction between positively charged drug-chitosan particles (D-NPs) and the SC-Ab complex. (**D**): Confocal image of CAD-NPs-FITC. Scale bar—11 µm.

Expression of mHSP70 on the tumor cell allows the use of the antibodies to HSP70 as a targeting molecule. Antibody 2E4 to C-fragment of HSP70 was selected as the targeting vector. Earlier, we synthesized furanoallocolchicinoid, a derivative of the antimitotic drug colchicine, containing a carboxyl group [30] (Figure 3A and Figure S1). This molecule was used as a model drug. 

The idea in the construction of the core–shell delivery system was based on hiding the drug inside the system and exposing the targeting antibody on its surface. The scheme of the synthesis is shown in Figure 3. The construction of core–shell NPs using positively charged biopolymer chitosan and its negatively charged derivative succinyl chitosan was described elsewhere [36]. We used standard quantities of polymers to form the particles. The main problem was incorporating the antibody to HSP70 into the delivery system. Pilot experiments were run to select the method for antibody binding to the polymer. It appeared that the EDC/NHS chemical binding of the antibody severely compromised the binding efficacy of the antibody. Finally, passive binding of the antibody to negatively charged succinyl chitosan was chosen (Figure 3B). Combining core particles and shell polymer with the immobilized antibody resulted in the complete core–shell particles (Figure 3B). For visualization, fluorescent dye FITC was incorporated at stage A after the incubation of chitosan with the drug. An overview of the particles is shown in Figure 3D.

### 3.4. Characterization of Core–Shell Delivery System Targeting mHSP70 C Fragment 

**Figure 4:**
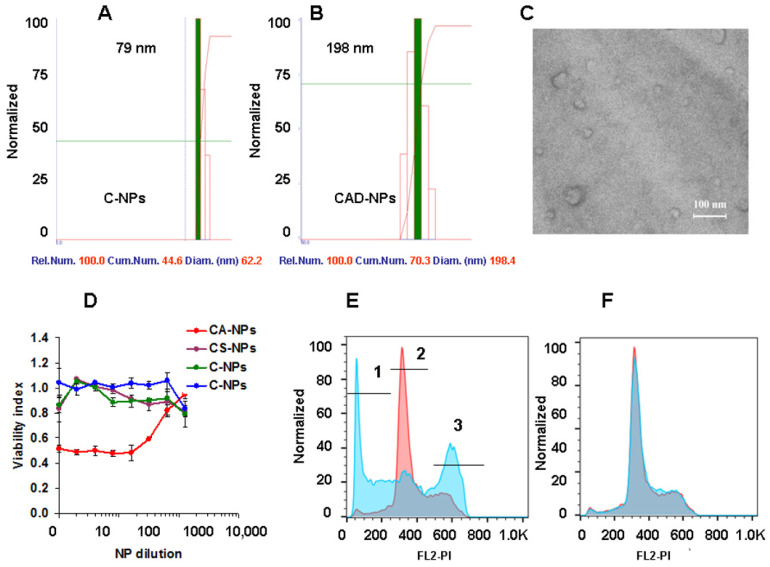
Characteristics of the delivery systems. (**A**,**B**): Dynamic light scattering of chitosan NPs (C-NPs) (**A**), chitosan-anti-HSP70-C fragment antibody drug conjugated NPs (CAD-NPs) (**B**); (**C**): SEM analysis of CAD-NPs. Scale 100 nm; (**D**): Viability assay of CSD-NPs, CS-NPs, and CA-NPs using PC-3 cells. (**E**,**F**): Cell cycle analysis of CD-NPs (**E**) and C-NPs (**F**) in PC-3 cells. CAD-NPs were used at 500-time dilution. Cells were incubated for 72 h and analyzed by flow cytometry. Numbers 1, 2, 3 correspond to apoptosis, G1, and G2/M cycles accordingly. Control cells are shown in pink and after the incubation with the samples—in blue.

The diameter of core nanoparticles formed by chitosan and core–shell NPs formed by chitosan, drug, succinoyl chitosan, and antibody or irrelevant murine Ig was estimated by DLS. The diameter of empty core NPs was 60–105 nm (Figure 4A, Table 2); inclusion of the drug did not change the diameter significantly, while covering the core NPs with succinylchitosan—antibody complex increased the diameter to 160–270 nm independently of which antibody was used (Figure 4B). SEM analysis demonstrated heterogenic particles smaller than 100 nm in diameter (Figure 4C). This is likely due to water loss when drying the particles for SEM. The polydispersity of NPs is shown in Table 2. The Zeta potential of the core and core–shell NPs was +25 ± 5 and −12 ± 8 mV, accordingly. Production of negatively charged delivery system is important as positively charged nano-, micro-particles can activate platelets and provoke thrombosis at intravenous injection.

The next aim was to analyze the functional activity of the drug incorporated into the core particle by chemical conjugation. The cytotoxic assay showed that the activity of the drug D was preserved (Figure 4D). Polymeric components and antibodies were nontoxic. The main mechanism of colchicine and its active derivatives is binding to tubulin. This means blocking mitotic spindle formation and cell division. This activity was analyzed by cell cycle in PC-3 cells. Only drug-containing NPs blocked PC-3 cells in the G2/M phase, as shown earlier [37], while C-NPs did not affect it (Figure 4E,F).

### 3.5. Characterization of 2E4 Antibody Activity in Core–Shell Delivery System 

**Figure 5:**
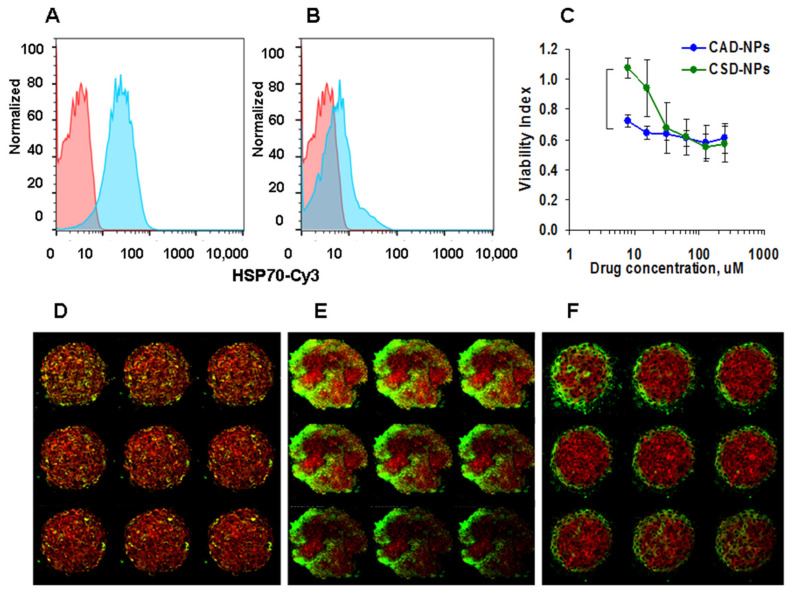
Specific binding and cytotoxicity of the delivery systems. (**A**,**B**): Flow cytometry of free 2E4 (**A**) and CA-NPs (**B**) binding to PC-3 cells. Control cells are shown in pink, and after the incubation with the samples, in blue. (**C**): Viability of PC-3 cells after incubation with antibody–drug conjugates CAD-NPs (blue) and control CSD-NPs (green). Data are shown as indices of proliferation. The statistical difference is shown with the bracket. (**D**–**F**): Confocal microscopy images of PC-3 3D cultures incubated 24 h with C-NPs (**D**), CAD-NPs (**E**), and CSD-NPs (**F**) stained with secondary antibody to murine IgG-FITC (green). Spheroids are stained with MitoTrackerRed.

Finally, it was important to estimate the binding activity of the 2E4 antibody after inclusion into the delivery system. This was fulfilled using PC-3 cells positive for mHSP70 expression. Including the antibody into the system partially decreased the binding activity, possibly due to the effect of polymers surrounding the antibody (Figure 5A,B). Analysis of the targeting effect of antibody to mHSP70 demonstrated that CAD-NPs were more toxic to tumor cells than CSD-NPs loaded with the irrelevant murine IgG (Figure 5C, blue versus green lines accordingly). The CSD-NPs not containing the antibody to mHSP70 were still toxic, which were shown in vitro and in vivo [30]. These results were studied in different cell lines with close results (Table 3).

Additionally binding activity of the delivery systems was analyzed by confocal microscopy in 3D cultures of PC-3 cells. Antibody penetration was studied by its staining using secondary anti-mouse IgG-FITC. Control C-NPs were not found in the cells (Figure 5D); CAD-NPs containing 2E4 antibody penetrated deeper into the 3D spheroid (Figure 5E), while CSD-NPs bound the cells but did not penetrate (Figure 5F).

Taken collectively, an efficient protocol of the delivery system to mHSP70-positive cells was developed, permitting the usage of antibodies to HSP70 as a targeting vector in antitumor therapy.

## 4. Discussion

Over the past decade, immunotherapy of oncological diseases using monoclonal antibodies against tumor-associated antigens has become increasingly widespread. Currently, several antibody-based companies are producing more than a dozen drugs for the treatment of cancer patients, and a large number of new antitumor therapeutic antibodies are in clinical trials [2]. The development of new approaches to antitumor immunotherapy is based on the ability of monoclonal antibodies against specific markers of cancer cells to induce not only antitumor responses associated with the direct interaction of the antibodies with malignant tissue but also cytostatic effects by blocking tumor cell growth. Cytostatic effect by blocking tumor cell receptors, receptor-mediated apoptosis of target cells, complement development and antibody-dependent cytotoxic antitumor immune response, and formation of long-term adaptive antitumor immune response are all possible mechanisms of this therapy [38]. Obviously, one of the most important factors in the development of new drugs for targeted immunotherapy using monoclonal antibodies is the search for biomarkers of malignant neoplasms, i.e., antigenic structures on the surface of cancer cells that are absent or characterized by a minimal level of expression in normal tissues. However, for many antigens considered to be tumor-associated membrane structures, this condition is only partially met. In most cases, tumor-specific antigens include molecules characterized by a significantly higher level of expression on the surface of cancer cells than on normal cells. However, the known biomarkers of cancer cells include a number of unique antigens that are not detectable in normal tissues but are expressed on the surface of tumor cells. These include several variants of heat shock proteins, including HSP70, which are present on the surface of many types of tumor cells. 

Chitosan possesses antimicrobial, mucoadhesive, wound healing, and immunomodulatory properties. Chitosan NPs have been extensively investigated for treating various diseases, including cancer [39,40,41]. High encapsulation efficiency, good stability in biological fluids, biodegradability, and biocompatibility make chitosan an attractive material to develop nanocarriers for tumor targeting therapy [41,42]. Different types of chitosan-based nanocarriers and core–shell NPs have been designed for the passive or active delivery of anti-cancer drugs [43,44,45,46,47]. Model drugs such as methotrexate, doxorubicin, and paclitaxel loaded onto chitosan carriers were delivered into tumor cells both in vitro and in vivo [48,49,50,51]. Among antibody-loaded chitosan nanocarriers, Lazer et al. suggested targeting the colon cancer cells using antibodies to doublecortin-like kinase 1 (DCLK1) [52]. Overexpression of DCLK1 is reported in colon, gastric, renal, pancreatic, and other cancers. Chitosan-folic acid-antibody NPs were prepared from Chitosan-Folic acid conjugate and antibody solution using TPP, as in our case. The authors demonstrated that such NPs induced apoptosis and inhibited the migration and invasion of colon cancer cells. Another example of targeting antibody-based chitosan NPs was presented by Kumar Mehata et al., who decorated NPs using Trastuzumab binding with human epidermal growth factor receptor type-2 (HER-2). Docetaxel-loaded chitosan NPs were in the range of 126–186 nm and showed an enhanced cellular uptake and cytotoxicity in HER-2 positive SK-BR-3 cells both in vitro and in vivo [53].

Recently, we showed that coating supermagnetic iron oxide nanoparticles (SPI-ONs) with antibodies against HSP70 enhanced their antitumor activity [54,55]. There is a possibility to combine targeting of surface HSP70 on tumor cells with the inhibition of its chaperone function in these cells [56]. 

In our model, we target epithelial tumor cells that express HSP70 on the surface, specifically in multi-cellular spheroids that mimic tumor growth in vivo. We have developed a complex core–shell delivery system targeting mHSP70. C-fragment of HSP70 was shown to be better exposed on the cell surface and can be recommended for targeting tumor cells than full-length or anti-N-fragment HSP70 antibodies. Using the anti-C-fragment HSP70 2E4 antibody, polymeric chitosan-based core–shell nanoparticles were developed. We showed that the chemical conjugation of colchicine derivative to chitosan did not interfere with the antimitotic activity of the drug. Such conjugation prevents the drug from leaking into the blood. Passive antibody loading onto succinylchitosan via electrostatic interaction was sufficient to deliver the drug to the HSP70-positive cells. However, other chemical conjugations can improve the functional activity of the delivery system (more antibodies can be loaded, less loss in blood, and longer storage achieved). 

The results obtained have demonstrated a reliable and significant targeting effect of anti-HSP70 monoclonal antibodies.

## Figures and Tables

**Table 1:** Structure of NPs used in this work.

NP Samples	1	2	3	4	5
Name	CAD-NPs *	CSD-NPs	CA-NPs	CS-NPs	C-NPs
Chitosan (core)	+	+	+	+	+
Succinoyl chitosan (shell)	+	+	+	+	+
Anti-HSP70 antibody	+	-	+	-	-
Murine IgG	-	+	-	+	-
Allocolchicinoid (drug)	+	+	-	-	-

* C—chitosan; A—mAb; D—allocolchicinoid; NPs—nanoparticles.

**Table 2:** Characteristics of NPs.

NP Samples	1	2	3	4	5
Parameters	CAD-NPs	CSD-NPs	CA-NPs	CS-NPs	C-NPs
Diameter, nm	199 +/− 60	198 +/− 60	181 +/− 50	179 +/− 60	80 +/− 20
Polydispersity (Rel. Var.)	0.30 +/− 0.05	0.12 +/− 0.08	0.06 +/− 0.02	0.21 +/− 0.15	0.03 +/− 0.05
Zeta potential, mV	−12 +/− 4	−14 +/− 5	−12 +/− 5	−15 +/− 4	+25 +/− 5
Protein, μg/mL	155 +/− 7	160 +/− 14	180 +/− 20	165 +/− 12	0
Drug, μM	501 +/− 30	500 +/− 42	0	0	0
Chitosan, mg/mL	2	2	2	2	2
Succinoyl chitosan, mg/mL	0.5	0.5	0.5	0.5	0.5

**Table 3:** Cytotoxicity (IC50) of different preparations.

		PC-3	BT-20	SW837	PC-3	BT-20	SW837
		Dilution			μM		
1	CAD-NPs	500 +/− 90	800 +/− 100	230 +/− 20	1.0 +/− 0.2	0.6 +/− 0.1	2.2 +/− 0.2
2	CSD-NPs	100 +/− 10	50 +/− 7	50 +/− 8	5.0 +/− 1	10.0 +/− 2.2	10.0 +/− 1.8
	**Ratio**				5.0	16.0	4.6
3	CS-Nps	<10	<10	<10	>2000	>2000	>2000
4	CA-NPs	<10	<10	<10	>2000	>2000	>2000
5	C-NPs	<10	<10	<10	>2000	>2000	>2000

## Data Availability

Data are contained within the article.

## Supplementary Materials

The following supporting information can be downloaded at: https://www.mdpi.com/article/10.3390/nano14231873/s1, Figure S1: Structures of chitosan (A), succinylchitosan (B) and allocolchicinoid (C).

## References

[1] Abès R., Teillaud J.L. (2011). Modulation of tumor immunity by therapeutic monoclonal antibodies. Cancer Metastasis Rev..

[2] Kierny M.R., Cunningham T.D., Kay B.K. (2012). Detection of biomarkers using recombinant antibodies coupled to nanostructured platforms. Nano Rev..

[3] Multhoff G. (2007). Heat shock protein 70 (Hsp70): Membrane location, export and immunological relevance. Methods.

[4] Stangl S., Gehrmann M., Riegger J., Kuhs K., Riederer I., Sievert W., Hube K., Mocikat R., Dressel R., Kremmer E. (2011). Targeting membrane heat-shock protein 70 (Hsp70) on tumors by cmHsp70.1 antibody. Proc. Natl. Acad. Sci. USA.

[5] De Maio A., Hightower L. (2021). The interaction of heat shock proteins with cellular membranes: A historical perspective. Cell Stress Chaperones.

[6] Tagaeva R., Efimova S., Ischenko A., Zhakhov A., Shevtsov M., Ostroumova O. (2023). A new look at Hsp70 activity in phosphatidylserine-enriched membranes: Chaperone-induced quasi-interdigitated lipid phase. Sci. Rep..

[7] Makky A., Czajor J., Konovalov O., Zhakhov A., Ischenko A., Behl A., Singh S., Abuillan W., Shevtsov M. (2023). X-ray reflectivity study of the heat shock protein Hsp70 interaction with an artificial cell membrane model. Sci. Rep..

[8] Lamprecht C., Gehrmann M., Madl J., Römer W., Multhoff G., Ebner A. (2018). Molecular AFM imaging of Hsp70-1A association with dipalmitoyl phosphatidylserine reveals membrane blebbing in the presence of cholesterol. Cell Stress Chaperones.

[9] Sugawara S., Kawano T., Omoto T., Hosono M., Tatsuta T., Nitta K. (2009). Binding of Silurus asotus lectin to Gb3 on Raji cells causes disappearance of membrane-bound form of HSP70. Biochim. Biophys. Acta.

[10] Gehrmann M., Liebisch G., Schmitz G., Anderson R., Steinem C., De Maio A., Pockley G., Multhoff G. (2008). Tumor-specific Hsp70 plasma membrane localization is enabled by the glycosphingolipid Gb3. PLoS ONE.

[11] Rosenzweig R., Nillegoda N.B., Mayer M.P., Bukau B. (2019). The Hsp70 chaperone network. Nat. Rev. Mol. Cell Biol..

[12] Joshi P., Garg S., Mani S., Shoaib R., Jakhar K., Almuqdadi H.T.A., Sonar S., Marothia M., Behl A., Biswas S. (2024). Targeting host inducible-heat shock protein 70 with PES-Cl is a promising antiviral strategy against SARS-CoV-2 infection and pathogenesis. Int. J. Biol. Macromol..

[13] Marothia M., Behl A., Maurya P., Saini M., Shoaib R., Garg S., Kumari G., Biswas S., Munjal A., Anand S. (2024). Targeting PfProhibitin 2-Hu-Hsp70A1A complex as a unique approach towards malaria vaccine development. iScience.

[14] Breuninger S., Stangl S., Werner C., Sievert W., Lobinger D., Foulds G.A., Wagner S., Pickhard A., Piontek G., Kokowski K. (2018). Membrane Hsp70-A Novel Target for the Isolation of Circulating Tumor Cells After Epithelial-to-Mesenchymal Transition. Front. Oncol..

[15] Ponomarev E.D., Tarasenko T.N., Sapozhnikov A.M. (2000). Splenic murine cytotoxic cells recognize surface HSP70 on culture-adapted EL-4 lymphoma cells. Immunol. Lett..

[16] Pockley A.G. (2001). Heat shock proteins in health and disease: Therapeutic targets or therapeutic agents?. Expert Rev. Mol. Med..

[17] Tsan M.F., Gao B. (2004). Cytokine function of heat shock proteins. Am. J. Physiol. Cell Physiol..

[18] Multhoff G. (2009). Activation of natural killer cells by heat shock protein 70. Int. J. Hyperthermia..

[19] Srivastava P. (2002). Roles of heat-shock proteins in innate and adaptive immunity. Nat. Rev. Immunol..

[20] Matzinger P. (2002). The danger model: A renewed sense of self. Science.

[21] Binder R.J., Vatner R., Srivastava P. (2004). The heat-shock protein receptors: Some answers and more questions. Tissue Antigens.

[22] van Eden W., van der Zee R., Prakken B. (2005). Heat-shock proteins induce T-cell regulation of chronic inflammation. Nat. Rev. Immunol..

[23] Prohászka Z., Singh M., Nagy K., Kiss E., Lakos G., Duba J., Füst G. (2002). Heat shock protein 70 is a potent activator of the human complement system. Cell Stress Chaperones.

[24] Arispe N., Doh M., Simakova O., Kurganov B., De Maio A. (2004). Hsc70 and Hsp70 interact with phosphatidylserine on the surface of PC12 cells resulting in a decrease of viability. FASEB J..

[25] Stangl S., Tei L., De Rose F., Reder S., Martinelli J., Sievert W., Shevtsov M., Öllinger R., Rad R., Schwaiger M. (2018). Preclinical Evaluation of the Hsp70 Peptide Tracer TPP-PEG24-DFO [89Zr] for Tumor-Specific PET/CT Imaging. Cancer Res..

[26] Aranaz I., Mengíbar M., Harris R., Paños I., Miralles B., Acosta N., Galed G., Heras Á. (2009). Functional characterization of chitin and chitosan. Curr. Chem. Biol..

[27] Wang W., Xue C., Mao X. (2020). Chitosan: Structural modification, biological activity and application. Int. J. Biol. Macromol..

[28] Prabaharan M. (2008). Review paper: Chitosan derivatives as promising materials for controlled drug delivery. J. Biomater. Appl..

[29] Tang W., Wang J., Hou H., Li Y., Wang J., Fu J., Lu L., Gao D., Liu Z., Zhao F. (2023). Review: Application of chitosan and its derivatives in medical materials. Int. J. Biol. Macromol..

[30] Svirshchevskaya E.V., Gracheva I.A., Kuznetsov A.G., Myrsikova E.V., Grechikhina M.V., Zubareva A.A., Fedorov A.Y. (2016). Antitumor Activity of Furanoallocolchicinoid-Chitosan Conjugate. Med. Chem..

[31] Hawash M. (2022). Recent Advances of Tubulin Inhibitors Targeting the Colchicine Binding Site for Cancer Therapy. Biomolecules.

[32] Lu L., Li K., Pu J., Wang S., Liang T., Wang J. (2024). Dual-target inhibitors of colchicine binding site for cancer treatment. Eur. J. Med. Chem..

[33] Deng S., Krutilina R.I., Hartman K.L., Chen H., Parke D.N., Wang R., Mahmud F., Ma D., Lukka P.B., Meibohm B. (2022). Colchicine-Binding Site Agent CH-2-77 as a Potent Tubulin Inhibitor Suppressing Triple-Negative Breast Cancer. Mol. Cancer Ther..

[34] Shagdarova B., Ilyina A.V., Lopatin S.A., Kartashov M.I., Arslanova L., Dzhavakhiya V., Varlamov V.P. (2018). Study of the protective activity of chitosan hydrolyzate against septoria leaf blotch of wheat and brown spot of tobacco. Appl. Biochem. Microbiol..

[35] Yamaguchi R., Arai Y., Itoh T., Hirano S. (1981). Preparation of partially N-succinylated chitosans and their cross-linked gels. Carbohydr. Res..

[36] Herdiana Y., Wathoni N., Shamsuddin S., Joni I.M., Muchtaridi M. (2021). Chitosan-Based Nanoparticles of Targeted Drug Delivery System in Breast Cancer Treatment. Polymers.

[37] Chaiputtanapun P., Lirdprapamongkol K., Thanaussavadate B., Phongphankhum T., Thippong T., Thangsan P., Montatip P., Ngiwsara L., Svasti J., Chuawong P. (2022). Biphasic Dose-Dependent G0/G1 and G2/M Cell-Cycle Arrest by Synthetic 2,3-Arylpyridylindole Derivatives in A549 Lung Cancer Cells. ChemMedChem.

[38] Dhodapkar K.M., Krasovsky J., Williamson B., Dhodapkar M.V. (2002). Antitumor monoclonal antibodies enhance cross-presentation ofcCellular antigens and the generation of myeloma-specific killer T cells by dendritic cells. J. Exp. Med..

[39] Al-Shadidi J.R.M.H., Al-Shammari S., Al-Mutairi D., Alkhudhair D., Thu H.E., Hussain Z. (2024). Chitosan nanoparticles for targeted cancer therapy: A review of stimuli-responsive, passive, and active targeting strategies. Int. J. Nanomed..

[40] Sachdeva B., Sachdeva P., Negi A., Ghosh S., Han S., Dewanjee S., Jha S.K., Bhaskar R., Sinha J.K., Paiva-Santos A.C. (2023). Chitosan nanoparticles-based cancer drug delivery: Application and challenges. Mar. Drugs.

[41] Wang H., Yu X., Su C., Shi Y., Zhao L. (2018). Chitosan nanoparticles triggered the induction of ROS-mediated cytoprotective autophagy in cancer cells. Artif. Cells Nanomed. Biotechnol..

[42] Jiang Y., Yu X., Su C., Zhao L., Shi Y. (2019). Chitosan nanoparticles induced the antitumor effect in hepatocellular carcinoma cells by regulating ROS-mediated mitochondrial damage and endoplasmic reticulum stress. Artif. Cells Nanomed. Biotechnol..

[43] Sun Y., Davis E. (2021). Nanoplatforms for targeted stimuli-responsive drug delivery: A review of platform materials and stimuli-responsive release and targeting mechanisms. Nanomaterials.

[44] Rahim M.A., Jan N., Khan S., Shah H., Madni A., Khan A., Jabar A., Khan S., Elhissi A., Hussain Z. (2021). Recent advancements in stimuli responsive drug delivery platforms for active and passive cancer targeting. Cancers.

[45] Lee S.J., Koo H., Jeong H., Huh M.S., Choi Y., Jeong S.Y., Byun Y., Choi K., Kim K., Kwon I.C. (2011). Comparative study of photosensitizer loaded and conjugated glycol chitosan nanoparticles for cancer therapy. J. Control Release.

[46] Lim C.K., Shin J., Kwon I.C., Jeong S.Y., Kim S. (2012). Iodinated photosensitizing chitosan: Self-assembly into tumor-homing nanoparticles with enhanced singlet oxygen generation. Bioconjug. Chem..

[47] Lee H.M., Jeong Y.I., Kim D.H., Kwak T.W., Chung C.W., Kim C.H., Kang D.H. (2013). Ursodeoxycholic acid-conjugated chitosan for photodynamic treatment of HuCC-T1 human cholangiocarcinoma cells. Int. J. Pharm..

[48] Al-Nemrawi N., Hameedat F., Al-Husein B., Nimrawi S. (2022). Photolytic controlled release formulation of methotrexate loaded in Chitosan/TiO2 nanoparticles for breast cancer. Pharmaceuticals.

[49] Choi Y., Han H., Jeon S., Yoon H.Y., Kim H., Kwon I.C., Kim K. (2020). Deep tumor penetration of doxorubicin-loaded glycol chitosan nanoparticles using high-intensity focused ultrasound. Pharmaceutics.

[50] Yoon K., Jung S., Ryu J., Park H.J., Oh H.K., Kook M.S. (2023). Redox-sensitive delivery of doxorubicin from nanoparticles of poly(ethylene glycol)-chitosan copolymer for treatment of drug-resistant oral cancer cells. Int. J. Mol. Sci..

[51] Gupta U., Sharma S., Khan I., Gothwal A., Sharma A.K., Singh Y., Chourasia M.K., Kumar V. (2017). Enhanced apoptotic and anticancer potential of paclitaxel loaded biodegradable nanoparticles based on chitosan. Int. J. Biol. Macromol..

[52] Lazer L.M., Kesavan Y., Gor R., Ramachandran I., Pathak S., Narayan S., Anbalagan M., Ramalingam S. (2022). Targeting colon cancer stem cells using novel doublecortin like kinase 1 antibody functionalized folic acid conjugated hesperetin encapsulated chitosan nanoparticles. Colloids Surf. B Biointerfaces.

[53] Kumar M.A., Bharti S., Singh P., Viswanadh M.K., Kumari L., Agrawal P., Singh S., Koch B., Muthu M.S. (2019). Trastuzumab decorated TPGS-g-chitosan nanoparticles for targeted breast cancer therapy. Colloids Surf. B Biointerfaces.

[54] Shevtsov M., Bobkov D., Yudintceva N., Likhomanova R., Kim A., Fedorov E., Fedorov V., Mikhailova N., Oganesyan E., Shabelnikov S. (2024). Membrane-bound heat shock protein mHsp70 is required for migration and invasion of brain tumors. Cancer Res. Commun..

[55] Kimm M.A., Shevtsov M., Werner C., Sievert W., Zhiyuan W., Schoppe O., Menze B.H., Rummeny E.J., Proksa R., Bystrova O. (2020). Gold Nanoparticle Mediated Multi-Modal CT Imaging of Hsp70 Membrane-Positive Tumors. Cancers.

[56] Du S., Liu Y., Yuan Y., Wang Y., Chen Y., Wang S., Chi Y. (2022). Advances in the study of HSP70 inhibitors to enhance the sensitivity of tumor cells to radiotherapy. Front. Cell Dev. Biol..

