# Supplementary figures and images for: Core–Shell Chitosan Particles Targeting Membrane-Bound Heat Shock Protein 70 for Cancer Therapy
Source: Nanomaterials (Basel). 2024 Nov 22;14(23):1873. doi: 10.3390/nano14231873 (PMC11643800; doi:10.3390/nano14231873)

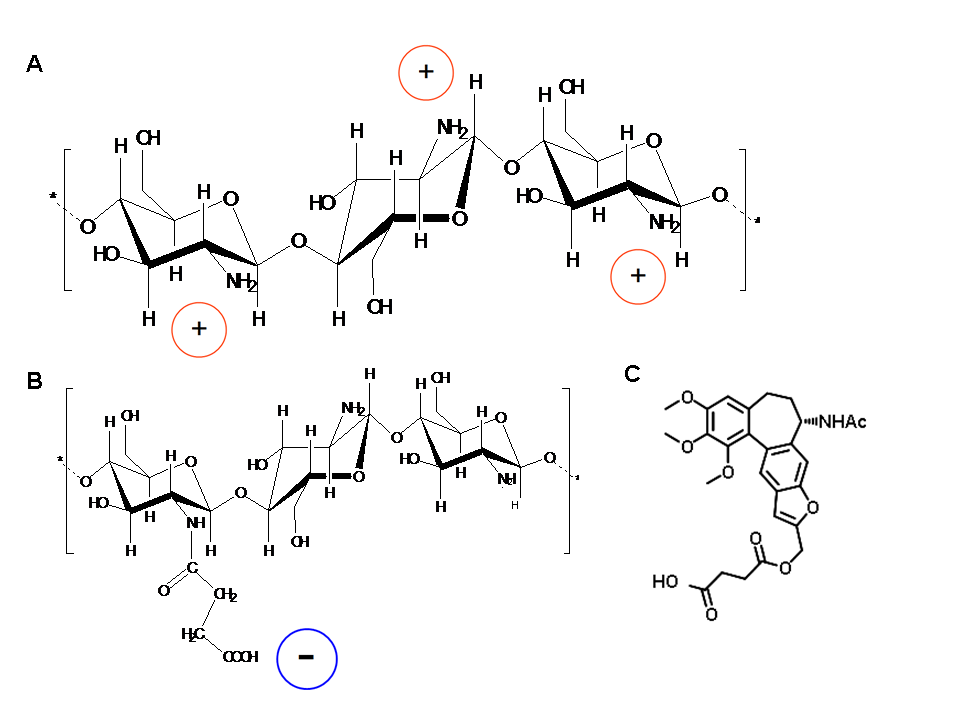


Figure S1. Structures of chitosan (A), succinylchitosan (B) and allocolchicinoid (C).

Supplement: Supplementary file 1 [file nanomaterials-14-01873-s001.zip › nanomaterials-3264394-supplementary.docx]
